# Standardization of definitions in focal therapy of prostate cancer: report from a Delphi consensus project

**DOI:** 10.1007/s00345-016-1782-x

**Published:** 2016-02-18

**Authors:** A. W. Postema, T. M. De Reijke, O. Ukimura, W. Van den Bos, A. R. Azzouzi, E. Barret, D. Baumunk, A. Blana, A. Bossi, M. Brausi, J. A. Coleman, S. Crouzet, J. Dominguez-Escrig, S. Eggener, R. Ganzer, S. Ghai, I. S. Gill, R. T. Gupta, T. O. Henkel, M. Hohenfellner, J. S. Jones, F. Kahmann, C. Kastner, K. U. Köhrmann, G. Kovacs, R. Miano, R. J. van Moorselaar, N. Mottet, L. Osorio, B. R. Pieters, T. J. Polascik, A. R. Rastinehad, G. Salomon, R. Sanchez-Salas, M. Schostak, L. Sentker, K. J. Tay, I. M. Varkarakis, A. Villers, J. Walz, J. J. De la Rosette

**Affiliations:** 1 Departments of Urology, AMC University Hospital, Amsterdam, The Netherlands; 2 Departments of Radiation Oncology, AMC University Hospital, Amsterdam, The Netherlands; 3 USC Institute of Urology, Keck School of Medicine, University of Southern California, Los Angeles, CA USA; 4 Department of Urology, Kyoto Prefectural University of Medicine, Kyoto, Japan; 5 Department of Urology, Angers University Hospital, Angers, France; 6 Department of Urology, Institut Montsouris, Université Paris Descartes, Paris, France; 7 Department of Urology, Magdeburg University Medical Center, Magdeburg, Germany; 8 Department of Urology, Fuerth Hospital, Fuerth, Germany; 9 Department of Radiation Oncology, Gustave Roussy Institute, Villejuif, France; 10 Department of Urology, Ospedale Civile Ramazzini, Carpi, Italy; 11 Department of Surgery, Memorial Sloan-Kettering Cancer Center, New York, NY USA; 12 Department of Urology and Transplantation, Edouard Herriot Hospital, Lyon, France; 13 Department of Urology, Instituto Valenciano de Oncología, Valencia, Spain; 14 Department of Urology, University of Chicago, Chicago, IL USA; 15 Department of Urology, University of Leipzig, Leipzig, Germany; 16 Joint Department of Medical Imaging, University Health Network, University of Toronto, Toronto, Canada; 17 Departments of Radiology, Duke University Medical Center, Durham, NC USA; 18 Departments of Surgery, Duke University Medical Center, Durham, NC USA; 19 Urologische Praxis Dr. Henkel & Dr. Kahmann, Berlin, Germany; 20 Department of Urology, University of Heidelberg, Heidelberg, Germany; 21 Glickman Urological and Kidney Institute, Cleveland Clinic, Cleveland, OH USA; 22 CamPARI Prostate Cancer Clinic, Cancer Directorate, Cambridge University Hospitals Trust, Cambridge, UK; 23 Department of Urology, Theresien Krankenhaus Mannheim, Mannheim, Germany; 24 Interdisciplinary Brachytherapy Unit, University of Lübeck, Lübeck, Germany; 25 Division of Urology, Department of Experimental Medicine and Surgery, University of Rome Tor Vergata, Rome, Italy; 26 Department of Urology, Free University Medical Centre, Amsterdam, The Netherlands; 27 Department of Urology, University Hospital St Etienne, Saint-Étienne, France; 28 Department of Urology, Porto Hospital Centre, Porto, Portugal; 29 Department of Urology, Hofstra North Shore-Lij, Hofstra University, Hempstead, NY USA; 30 Martini-Clinic Prostate Cancer Center, University Hospital Hamburg-Eppendorf, Hamburg, Germany; 31 Urologische Gemeinschaftspraxis, Sinsheim, Germany; 32 2nd Department of Urology, Athens Medical University, University of Athens, Athens, Greece; 33 Department of Urology, Lille University Medical Center, Lille, France; 34 Department of Urology, Institut Paoli-Calmettes, Marseille, France

**Keywords:** Focal therapy, Prostate cancer, Consensus, Definitions, Standardization, Outcome

## Abstract

**Purpose:**

To reach standardized terminology in focal therapy (FT) for prostate cancer (PCa).

**Methods:**

A four-stage modified Delphi consensus project was undertaken among a panel of international experts in the field of FT for PCa. Data on terminology in FT was collected from the panel by three rounds of online questionnaires. During a face-to-face meeting on June 21, 2015, attended by 38 experts, all data from the online rounds were reviewed and recommendations for definitions were formulated.

**Results:**

Consensus was attained on 23 of 27 topics; *TargetedFT* was defined as a lesion-based treatment strategy, treating all identified significant cancer foci; *FT* was generically defined as an anatomy-based (zonal) treatment strategy. Treatment failure due to the ablative energy inadequately destroying treated tissue is defined as *ablation failure.* In *targeting failure* the energy is not adequately applied to the tumor spatially and *selection failure* occurs when a patient was wrongfully selected for FT. No definition of biochemical recurrence can be recommended based on the current data. Important definitions for outcome measures are potency (minimum IIEF-5 score of 21), incontinence (new need for pads or leakage) and deterioration in urinary function (increase in IPSS >5 points). No agreement on the best quality of life tool was established, but UCLA-EPIC and EORTC-QLQ-30 were most commonly supported by the experts. A complete overview of statements is presented in the text.

**Conclusion:**

Focal therapy is an emerging field of PCa therapeutics. Standardization of definitions helps to create comparable research results and facilitate clear communication in clinical practice.

**Electronic supplementary material:**

The online version of this article (doi:10.1007/s00345-016-1782-x) contains supplementary material, which is available to authorized users.

## Introduction

Prostate cancer (PCa) is traditionally treated with whole-gland treatments such as radical prostatectomy, whole-gland external beam radiotherapy and whole-gland brachytherapy [1]. Active surveillance allows selected patients with low-risk PCa to postpone or avoid radical treatment and the associated risk of toxicity [1]. Focal therapy (FT) is a fairly recent and rapidly developing field of PCa treatment where only a portion of the prostate gland is treated. Focal therapy intends to strike a balance between treating what must be treated while minimizing toxicity [2]. Different ablative energies employed for FT and under investigation include: cryosurgery, high-intensity focused ultrasound (HIFU), irreversible electroporation (IRE), laser ablation therapy, photodynamic therapy, and brachytherapy [3]. In the accumulating literature on FT, different terminology is used for different variants of tissue-sparing treatments, targeted lesions, and oncologic, functional and procedural outcomes. Standardization in definitions will aid in creating comparable research results in the literature and with clear communication in clinical settings. To achieve widely recognized standardized terminology in FT, we conducted an international multidisciplinary consensus project.

## Methods

The Delphi method is a widely accepted method to achieve consensus among experts and is employed in economics, politics, military decision making and medicine [4]. The basis of the Delphi method is that a panel of experts is repeatedly presented a series of questions. Each successive round the question and answer possibilities are modified based on the responses to the previous round. The anonymous aggregated results and comments of the previous round are presented to the panel, allowing the participants to reassess their opinion. The intended outcome is a convergence of opinions with a minimized effect of peer-pressure and dominant individuals influencing group choices.

**Fig. 1:**
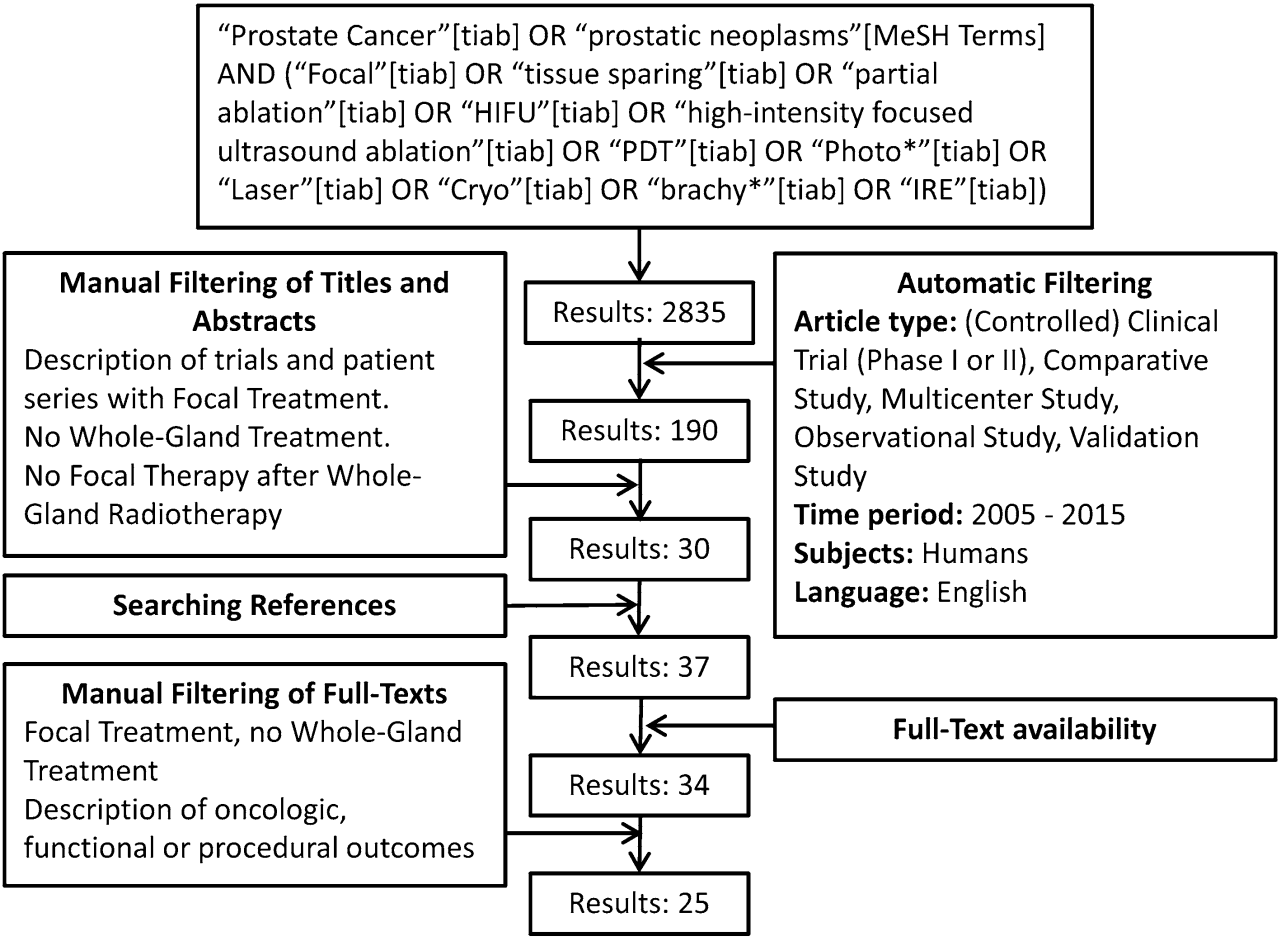
Systematic search

A systematic literature search of the English literature was conducted on “prostate cancer”, “focal therapy” and the various FT modalities. The initial search yielded 190 results with subsequent automatic filtering, screening of titles, abstracts and full-texts resulting in the selection of 25 papers for data extraction. The search term and results are provided in Fig. 1. A group of 113 experts was invited to participate on the basis of the literature search and peer recommendation.

From the papers identified by the systematic search, various definitions of oncologic, functional and procedural outcomes were extracted and these data formed the basis of the questionnaires. The questionnaires constructed were presented to the participants in three successive rounds between May 15 and June 16, 2015 (using www.surveymonkey.com). The level of agreement necessary to achieve consensus was set at 80 %. During the 8th International Symposium on Focal Therapy and Imaging in Prostate & Kidney Cancer (www.focaltherapy.org), a face-to-face meeting was held among 38 of the experts. All results from the online questionnaires were presented and discussed. Topics on which the online panel had achieved consensus were not overturned. Topics on which consensus had not been achieved were discussed in detail. Recommendations for definitions were formulated on all topics. An overview of the results of the online rounds and final recommendations for definitions is presented in Addendum 2.

## Results

### Systematic search

Twenty-five reports of clinical trials and trial protocols describing oncological, functional and procedural outcomes after a focal treatment, published after 2005, were identified by the systematic search. The modalities described were cryoablation [5–12], HIFU [6, 13–17], IRE [6, 13–21], laser ablation therapy [22–24], VTP [6, 25–28] and brachytherapy [6, 29].

#### Treatment efficacy

Commonly reported measures of treatment efficacy are prostate-specific antigen (PSA) dynamics after treatment [5–17, 20–22, 25, 26, 28, 29], the presence of suspicious areas on follow-up MRI [13, 14, 16, 18, 20–22, 25] and follow-up biopsy results. The planned follow-up biopsy scheme varies: in some protocols both the treated zone and the untreated zone are biopsied in every patient [5, 7–11, 15, 17, 19, 25–27, 29]. In other protocols, only the treated zone is biopsied [22, 28], although some groups add biopsies from the untreated zone if new lesions are found on imaging [13, 14, 21]. Lee et al. [24] biopsied the ablated zone only at 3 months and performed a 12-core systematic biopsy and additional ablated zone biopsies at 1 year. Dickinson et al. [16] described treated zone biopsies at 1 year and template mapping biopsies at 3 years. Few authors define treatment success and failure, and the definitions vary: Ellis et al. [8] described an argument for only considering cancer found at follow-up in the treated zone or development of metastatic disease as treatment failure. Lindner et al. [27] referred to their four cases with residual tumor in the treated zone as treatment failures. Azzouzi et al. [25] defined a negative follow-up biopsy of the targeted zones as treatment success. In their protocol, Dickinson et al. [16] determined cancer control as the primary outcome using two definitions: (1) the combination of being free of any cancer in the treated zone and free of significant cancer in untreated zones at 3-year template biopsy or (2) free of significant disease in both treated and untreated zone at 3-year template biopsies. Onik et al. [11] proposed to define the combination of stable PSA and negative follow-up biopsy as a successful result. Durand et al. [7] defined treatment failure as positive follow-up biopsy in the treated lobe or biochemical failure according to the Phoenix criteria of PSA nadir +2 ng/mL at 18 months.

#### Trifecta

Trifecta as a measure for urinary continence, potency and cancer control is defined by three papers, although two different definitions were used: pad-free, leak-free continence, erections sufficient for penetration and no high volume disease or Gleason ≥7 disease on follow-up biopsies were used in two papers [13, 21]. Ahmeds’ et al. [14] definition differed by substituting a radiologic outcome for the biopsy outcome of using no evidence of clinical disease on MRI at 12 months as a surrogate for cancer control.

#### Significant cancer

All papers that defined clinically significant disease at biopsy agreed that Gleason ≥7 is significant at any cancer core length. The maximum length of Gleason 6 that is considered insignificant is 2 mm [14] or 3 mm [13, 16, 20, 21].

#### Radiographic failure

Several authors used imaging to determine whether residual tumor in the treated zone or new tumor outside the treated zone was present. Six papers reported the use of MRI [13, 14, 21, 22, 24, 29] and one additional paper [20] also defined criteria for suspicion of residual cancer: an early enhancing focus on the dynamic contrast sequence and residual restricted diffusion in the treatment area. Doppler ultrasound was used to target suspicious lesions post-treatment in one study [5]. One protocol described using both MRI and transrectal ultrasonography at 1, 3 and 5 years post-treatment without stating the purpose of these investigations [18].

#### Biochemical status

Baseline and post-treatment PSA dynamics are reported by most papers [5–22, 24–29]. The more specific “biochemical recurrence” is reported by few papers and different definitions were used: The original ASTRO definition of three consecutive rises above the nadir was used by five authors [5, 8, 10, 11, 17]. Durand et al. [7] used the Phoenix definition of PSA nadir +2 ng/mL. Lambert et al. [9] described two conditions, meeting either one was considered biochemical recurrence: PSA nadir +2 ng/mL or a PSA nadir of less than 50 % of the pre-treatment level. Nguyen et al. [29] advocated an alternative definition of biochemical recurrence where two conditions should both be met: PSA nadir +2 ng/mL and a PSA velocity over 0.75 ng/mL.

#### Sexual function

Sexual function is most commonly reported using the International Index of Erectile Function 5 item version (IIEF-5), alternatively known as the Sexual Health Inventory for Men [6, 7, 15, 18, 19, 22, 24, 25, 27]. Durand et al. [7] defined an IIEF-5 score between 21 and 25 regardless of oral medication use as potent. The 15 item version (IIEF-15) was used by four authors [6–8, 10]. Two papers used the Brief Male Sexual Function Inventory BMSFI [5, 28, 30]. Six papers did not report the use of a validated questionnaire but did state the proportion of patients who were potent following FT [8–12, 20]. Four papers did not report sexual function [17, 23, 26, 29]. Different definitions for potency were in use besides the standardized questionnaires: the combination of achieving erections sufficient for vaginal penetration and being satisfied with sexual functioning regardless of the use of oral medication [10, 11], erections sufficient for vaginal penetration allowing phosphodiesterase-5 inhibitors [8, 14], erections sufficient for vaginal penetration without stating whether medication is allowed [9, 13, 20, 21], and the ability to maintain an erection when stimulated with or without oral medication [5]. However, many papers that reported potency as an outcome did not state how it is defined [6, 12, 15, 16, 18, 19, 22, 24, 25, 27, 28]. Durand et al. [7] reported the number of patients who are sexually active before and after treatment without providing a definition.

#### Urinary function

The most commonly used measure of urinary function was the IPSS [6, 7, 13–19, 21, 22, 24, 25, 27, 28]. The UCLA-EPIC urinary domain was used by six papers [13, 14, 16, 17, 19, 21, 24]. Continence was reported by several authors without providing an exact definition [6, 9, 12, 23, 24]. Commonly used definitions for incontinence were: the use of pads [7, 10, 11, 14, 20], urinary leakage [5, 8] or combined: urinary leakage or pads [13, 14, 21]. One paper described the use of the International Continence Society questionnaire [15].

#### Bowel function

The UCLA-EPIC bowel domain was mentioned by two papers [16, 21]. Other authors simply stated the absence of fistula, rectal discomfort, rectal bleeding, and change in frequency [14, 20, 22]. No definition for bowel toxicity in the context of FT was provided.

#### Quality of life

Quality of life (QoL) was generally reported based on standardized questionnaires. The UCLA-EPIC [13, 14, 16, 17, 19, 21, 24, 31] and FACIT (FACT-P and FACT-G) [13, 14, 16, 18, 21, 32, 33] were most prevalent in the 25 papers identified by the literature search. Other questionnaires used were MAX-PC [16, 21, 34], EORTC-QLQ-30 [15, 35], PORPUS [27, 36] and EQ-5D [16, 21, 37]. No specific definitions or cut-off values for the questionnaires were provided.

#### Complications

For the timing and severity of complications, several definitions and systems were used. Perioperative or periprocedural complications were reported but not exactly defined by three papers [19, 22, 27]. Many authors used either the Clavien–Dindo score [6, 7] or the Common Terminology Criteria for scoring Adverse Events (CTCEA) scoring system to grade adverse events [19, 20, 22, 25]. A CTCEA grade 3 and above and Clavien grade 3a and above were considered “severe”. Moore et al. [28] described minor complications without providing a definition [28].

#### Procedural outcomes

Procedure time was reported by five papers and two did not provide a precise description [7, 13, 23]. Oto et al. [22] reported the total time the patient spends in the MR unit. Azzouzi et al. [25] reported the procedure time including anesthesia, targeting and ablation. Most papers stated how many days the urethral catheter was left in situ following FT [6–8, 12, 17–19, 22, 23, 27, 28]. Many authors provided the time the patient remained in the hospital after FT [6–8, 13, 14, 18–20, 22, 23, 27]. Dickinson et al. [16] implied that treatment in day-care per definition does not involve an overnight stay.

### Consensus project: response rates and participants

One hundred thirteen experts were invited to participate. The response rate was 59 % (67/113) for the first round, 53 % (60/113) for the second and 65 % (73/113) for the third round. Complete personal details were collected during the last online round and showed the following participant backgrounds: 75 % were urologists, 11 % radiologists, 4 % radiotherapists, 4 % researchers, 3 % pathologists and 3 % medical oncologists. The average number of patients treated with FT annually reported by the panelists was 10–50 (47 %), 7 % of participants treated over 100 patients a year with FT. Cryosurgery, HIFU and brachytherapy were the most commonly used treatment modalities among the panel with 48, 51 and 29 % of the panelists reporting experience with these techniques. All consensus statements are summarized in Table 1. All registered participants to the online rounds and results of the online rounds discussed at the meeting are made available through Appendices 1 and 2, respectively.

**Table 1:** Definitions and consensus statements

Definition	Consensus statement regarding definition
*General definitions*	
Focal therapy (FT)	An anatomy-based (zonal) treatment strategy (e.g. targeting a quadrant, a lobe or both lobes sub-totally)
Targeted FT	A lesion-based focal treatment strategy targeting the identified tumors plus a safety margin
The aim of (targeted) FT for PCa	Eradication of all significant cancer(s)
Subtotal ablation	Any ablation where less than the whole gland is treated
Extended-hemiablation	An ablation where one lobe is completely treated plus a margin of the other lobe regardless of shape
Index lesion	The single dominant lesion in terms of grade and size where grade is more important. There can be only 1 index lesion. The term index lesion itself may be of limited use in the context of FT. It is more important to have an overview of all significant lesions that warrant treatment rather than a single defined index lesion
Salvage FT	Salvage FT refers to the situation where FT is applied to the prostate after whole-gland therapy, or in the same region of the prostate as previous FT. The prostate gland has to be in place
*Success and failure in focal therapy*	
Ablation failure	Ablation failure is a failure of the technique to destroy the tissue in the treated zone, evidenced by tumor found within the treated zone. Ablation failure is just one of the causes that can lead to failure of FT as a whole. Other types of failure include targeting failure and selection failure. Must be confirmed by targeted biopsy
Radiographic suspicion of ablation failure	A suspicion on imaging of tumor presence within the treated zone. mpMRI a suitable imaging modality to determine ablation failure
Residual disease	Cancer remaining in the target zone after FT
Selection failure	FT was inappropriately indicated, evidenced by short-term post-treatment identification of metastatic or locally advanced disease. There is no agreement on whether significant PCa in short-term biopsies taken inside or outside the treatment zone and the need for whole-gland treatment during follow-up constitute selection failure
Biochemical progression after targeted FT	PSA is the best marker to monitor the disease after targeted FT. However, there is currently no data on how to use PSA, i.e. there is no data to support any of the definitions for biochemical recurrence in the context of (targeted) FT
Pathological progression	An increase in Gleason score or tumor volume evidenced by a larger number of positive biopsies or larger per-core tumor involvement
*Baseline and outcome functional measures*	
Functional success of FT	The maintenance of voiding pattern, erectile function and quality of life assessed after 12 months
Erectile function	A qualitative definition of impotency exists: the persistent inability to attain and maintain an erection sufficient for satisfactory sexual performance. For reporting research the panel recommends defining significant erectile dysfunction using the IIEF-5 score <21, determined at 1 year
Sexually active	Patient-reported regular sexual activity
Urinary incontinence	The need to use pads or patient-reported leakage. More comprehensive data could be gathered by requesting patients to complete a micturition diary including the parameters: number of pads, leakage and urge
Significant deterioration of urinary function	An increase in IPSS >5 points
Quality of life	A quality-of-life questionnaire should be administered and both the UCLA-EPIC and the EORTC-QLQ-c-30 tools can be used although neither one is validated for the specific context of focal therapy
Bowel toxicity/GI side effects	The occurrence of: a change in stool frequency, fistula formation, soiling and/or blood in the stool after FT should constitute bowel toxicity/GI side effects. There is no consensus on whether mucus in the stool should also be included. The use of one of the existing grading systems for bowel toxicity is recommended
Intraoperative complications	Complications that cause damage to the patients’ health or require intervention to prevent damage
Short-term side effects	Side effects within 90 days of the procedure
Serious side effects	Clavien–Dindo-scale with 3 or greater as “serious” side effects
*Procedural outcomes*	
Procedure time	From the completion of anesthetic induction until the treating physician is finished
Hospital stay	The time from admittance until discharge
FT in day-care	Admittance, treatment and discharge on the same day
Catheterization time	The time from inserting the catheter until its removal, including time spent on the OR and the recovery-unit

All statements were accepted with >80 % consensus unless stated other otherwise

#### General definitions

The panel recommends defining “targeted focal therapy” as a lesion-based focal treatment of the target lesion(s) plus a safety margin. Ablating a quadrant, a lobe (hemiablation) or both lobes sub-totally would be defined as “focal therapy”. Other variants of these anatomy-based focal therapy templates are subtotal ablation (any ablation where less than the whole gland is treated) and extended hemi-ablation (an ablation where one lobe is completely treated plus a margin of the other lobe, regardless of shape). The aim of targeted focal therapy should be the eradication of all identified significant tumors. There was agreement the index lesion is the single dominant lesion in terms of grade and size; grade being more important. Although there was agreement that there can be only 1 index lesion, the term “index lesion” itself may be of limited clinical use in the context of FT. When there are multiple significant lesions, it is more important to have an overview of all lesions that require treatment rather than a single defined index lesion.

There was no consensus on the definition of salvage focal therapy during the online rounds. After discussion at the meeting, the panel supported the following statement: Salvage focal therapy refers to a situation where focal treatment is applied to the prostate after previous whole-gland therapy, or in the same region of the prostate as a previous FT. The prostate gland has to be in place.

#### Success and failure in focal therapy

In defining success and failure of FT, two levels were observed by the panel during the meeting: Failure of the focal treatment as a whole and several reasons for a focal treatment to fail (“ablation failure”, “targeting failure” and “selection failure”). Ablation failure is a failure of the technique to destroy the tissue within the intended treated zone, evidenced by tumor detected within the treated zone. Ablation failure must be confirmed histologically. Any cancer left in the treated zone only is termed “residual disease”. Radiographic suspicion of ablation failure is imaging suspect for tumor presence within the treated zone. The panelists unanimously agreed that multiparametric (mp) MRI is a suitable imaging tool for monitoring this, followed by 20 % for CEUS and 16 % for PET. Targeting failure occurs when the ablative energy is not correctly applied to the tumor spatially and for selection failure, FT was inappropriately indicated to the patient. The panel agreed that selection failure was definitely evidenced by short-term post-treatment identification of metastatic or locally advanced disease. However, during the meeting there was no agreement on how selection failure could be inferred from post-treatment biopsy results. There was agreement that PSA is the best marker to monitor disease activity after (targeted) FT, even though there is currently no data on how to use PSA after focal treatments where (malignant) prostate tissue is left in place. Therefore, there is no data to support any of the definitions for biochemical recurrence commonly used after whole-gland treatment in the context of (targeted) FT. In defining pathological progression the panel agrees that both an increase in Gleason score and tumor volume evidenced by a growing number of positive biopsies or larger per-core tumor involvement should be taken into consideration.

#### Baseline and outcome functional measures

The panel recommended defining functional success of focal therapy as the maintenance of voiding pattern, erectile function and QoL assessed after 12 months. In defining potency, most of the online panel agreed a minimum IIEF score should be used. During the face-to-face meeting, it was stressed that a widely endorsed qualitative definition of impotency already exists: “the persistent inability to attain and maintain an erection sufficient for satisfactory sexual performance”. However, a quantitative definition is useful in reporting and this quantitative definition should be based on the IIEF-5. The panel recommended defining significant erectile function following FT using the IIEF-5 score with a cut-off value of 21, determined at 1 year. The definition of sexually active should be based on patient-reported sexual activity.

Urinary incontinence is defined as the need to use pads or patient-reported leakage. During the face-to-face meeting, it was suggested that more comprehensive data should be gathered by requesting patients to fill in a voiding diary including: number of pads, leakage and urgency. The recommended definition of significant deterioration of urinary function is an IPSS increase >5 points.

In defining the QoL status of patients before and after FT different tools can be used. None of the tools were supported by more than 80 % of the panelists. The EORTC-QLQ-C-30 was supported by 75 % of the panel and the UCLA-EPIC by 70 %. During the face-to-face meeting, there was agreement that a QoL questionnaire should be although neither one is validated in the specific context of FT.

In defining “bowel toxicity” following FT, the panel agreed that a change in stool frequency, fistula formation, or the occurrence or increase of soiling and blood in the stool after FT constitutes bowel toxicity. There is no consensus on whether the occurrence or increased production of mucus in the stool should be included. During the face-to-face meeting, a recommendation was formulated to use either one (CTCEA or RTOG/EORTC) of the existing grading systems for bowel toxicity. There was agreement that it may not be necessary to present a full questionnaire to all patients but only to those who have indicated that they have gastro-intestinal side effects.

The recommended definition of short-term side effects is: side effects that become apparent within 90 days after the procedure. The panel recommended including only complications to the definition of intraoperative complications that cause damage to the patients’ health or require a subsequent intervention. Therefore, technical difficulties with the equipment and targeting difficulties due to anatomy are not necessarily intraoperative complications. The panel agreed to grade side effects using the Clavien–Dindo-scale regarding a grade 3 and higher as “serious side effects”.

#### Procedural outcomes

The panel recommended defining procedural time as the time period starting after the anesthetic induction is completed and the treating physician can start until the treating physician has finished the treatment. Hospital stay should be defined as the time from admission until discharge. The definition of FT in day-care should be: admittance, treatment and discharge on the same calendar day. The definition of catheterization time should be the time from inserting the catheter until its removal, including time spent on the OR and the recovery-unit.

## Discussion

The results of this Delphi consensus project will aid researchers in reporting FT outcomes in a standardized fashion, thereby allowing comparison and also facilitating clear communication among patients, clinicians and researchers. As standardized definitions can only succeed if they are widely endorsed by the experts in the field, a large-group consensus project is the ideal way to achieve this. Consensus projects can be a valuable tool, especially in fields such as FT where the clinical evidence is still building. It is therefore not surprising that several consensus projects on various aspects of FT have been undertaken in the past years. The topics handled in our consensus project partly overlap with the topics of other consensus meetings. There appears to have been a shift in the perceived aim of FT. The 2009 consensus project described by De la Rosette et al. [38] stipulated that the aim of FT is to eradicate all known prostate cancer while preserving uninvolved tissue, sparing genitourinary function. The 2010 consensus project described by Ahmed et al. [39] stated the aim of FT is to treat cancer and leave benign prostate and surrounding normal structures. They do not note the possibility to also leave insignificant disease untreated. Five years later, we formulated the aim of FT to be the eradication of all identifiable significant tumor(s). The consensus project described by Donaldson et al. [40] stated that FT should be the treatment of the dominant lesion or index lesion. They add that quadrant ablation could be a FT strategy but with lower level of consensus than lesion ablation only. This may imply their panel considered FT as a lesion-based technique, similar to our definition of targeted FT. Consistent with the concerns regarding secondary (non-index) lesions harboring clinically significant disease with metastatic potential formulated by Reis et al. [41], the participants of our consensus project underlined the necessity to find and treat all clinically significant cancer foci, regardless of the precise definition of the index lesion.

The consensus projects described by Muller et al. [42] and De la Rosette et al. [38] provided definitions for oncologic success and oncologic efficacy, respectively, that are similar to our definition of ablation success: negative biopsies in the treated area. The definitions around the concepts of success and failure provided by the consensus project described by Van den Bos et al. [43] differed slightly from ours; they recommended defining in-field failure as: (1) higher Gleason grade disease in the treated zone, (2) persistent cancer of similar or lower grade after repeat FT of the same area and (3) the need for additional treatment besides FT because of objective findings elsewhere in the gland. While the first two conditions are concordant with our definition of ablation failure, the latter does not. Finding tumor for which FT is not suitable during short-term follow-up elsewhere in the gland would constitute selection failure by our definition. Their definition of selection failure appears not to be related to adequate patient selection for FT but to the selection of tumor foci for treatment.

The project described by Muller et al. [42] described a definition of functional success comparable to ours: the absence of functional change in erectile function and ejaculatory function at 24 months, QoL at 24 months and urinary function at 12 months. The main difference is that present consensus does not include ejaculatory function and only uses the 12-month time point for all three domains of functional outcomes.

The consensus projects by Muller et al. [42] and Van den Bos et al. [43] provided recommendations for follow-up after FT. Similar to our results, they recommended to monitor PSA, although they also found no standardized definition of biochemical recurrence could be recommended. Other follow-up parameters include urinary function, sexual function and quality of life using standardized tools, including IPSS and IIEF questionnaires. In the reviewed literature, FACIT (FACT-P) and (FACT-G) and UCLA-EPIC questionnaires were the most prevalent tools for the assessment of QoL following FT. Muller et al. [42] could not achieve consensus on which QoL tool to be used, but the FACT-P was widely supported. In the project described by Van den Bos et al. [43], the UCLA-EPIC was recommended, a least for pre-treatment assessment of QoL. De la Rosette et al. [38] also recommended to use the FACT-P and EORTC questionnaire among several other options. In our project the EORTC-QLQ-c30 was widely endorsed besides the UCLA-EPIC, while the FACIT questionnaire was considered useful by only 50 % of our online panel. MpMRI is universally recommended as a tool for follow-up after FT, which is consistent with our panel agreeing unanimously that mpMRI is a suitable tool to define radiographic suspicion of ablation failure.

Several considerations regarding our consensus project are: as the results are based on expert opinion they amount to an Oxford Centre of Evidenced Based Medicine level 5 of evidence [44]. The group of experts we invited may not be representative of the larger medical community as their involvement in FT makes them more likely to be enthusiasts. Furthermore, the response rates to our online questionnaires were between 59 and 65 %, although this is likely caused by our attempt to invite a large and broad group of experts, further bias in our results might be caused by “non-believers” not participating. Although a total of 38 experts joined the face-to-face meeting, which took place at a FT meeting, their opinions may be more prominently represented in the final recommendations than the opinions of the participants of the online rounds only. The repetitive formulation and reformulation of questions and answer possibilities by the project leaders may also be a source of bias and can be seen as an inevitable limitation of the Delphi method.

Several important topics remain undefined because of consensus: (1) there was insufficient data (2) unresolved diverging opinions within the panel or (3) the topics were un-addressed by the current project. Examples of important unresolved matters are: “What constitutes a clinically significant tumor focus?”, “How should PSA dynamics be interpreted after FT?”, “How can QoL best be assessed after FT” and “What are adequate safety margins to be observed for the different treatment modalities?”. These topics can be addressed in future consensus projects. As the data on FT accumulates, it should become possible to formulate stronger recommendations and data-based guidelines on how best to research, apply and report focal treatments.

## Conclusion

Focal therapy is a rapidly evolving field of prostate cancer treatments that intends to prevent or delay whole-gland treatment associated morbidity without compromising oncologic safety for a large group of patients. For the development and implementation of these treatments, it is important to have standardized reporting criteria. The current consensus project provides recommendations for standardized definitions endorsed by a wide group of experts in the field.

## Electronic supplementary material

Below is the link to the electronic supplementary material.

Supplementary material 1 (PDF 215 kb)

Supplementary material 2 (PDF 326 kb)
