# Supplementary material for: Standardization of definitions in focal therapy of prostate cancer: report from a Delphi consensus project
Source: World J Urol. 2016 Feb 18;34(10):1373–82. doi: 10.1007/s00345-016-1782-x (PMC5026990; doi:10.1007/s00345-016-1782-x)
Supplement: Supplementary file 1 — Supplementary material 1 (PDF 215 kb) [file 345_2016_1782_MOESM1_ESM.pdf]

Addendum 1. All registered participants to the online rounds.

Not all participants were registered as some responded anonymously.

Name	Country	Name	Country
Andre de Castro Abreu	USA	Badrinath Konety	USA
Hashim Ahmed	UK	Györge Kovács	Germany
Gerald Andriole	USA	Pilar Laguna Pes	The Netherlands
Eduard Baco	Norway	Massimo Lazzeri	Italy
Duke Bahn	USA	Raymond Leiveillee	USA
Chris Bangma	The Netherlands	Yoh Matsuoka	Japan
Eric Barret	France	Roberto Miano	Italy
Daniel Baumunk	Germany	Caroline Moore	UK
Andreas Blana	Germany	Rodolfo Montironi	Italy
Alberto Bossi	France	Jeroen van Moorselaar	The Netherlands
Simon Bott	UK	Satoru Muto	Japan
Maurizio Brausi	Italy	Sacha Pahernik	Germany
Jeffrey Cadeddu	USA	Bradley Pieters	The Netherlands
Xavier Cathelineau	France	Thomas Polascik	USA
Jonathan Coleman	USA	Ardeshir Rastinehad	USA
Sebastien Crouzet	France	Theo de Reijke	The Netherlands
Mihir Desai	USA	John Rewcastle	USA
Jose Dominguez-Escrig	Spain	Jean de la Rosette	The Netherlands
Vinay Duddalwar	USA	Dilara Savci-Heijink	The Netherlands
Scott Eggener	USA	Rafael Sanchez-Salas	France
Behfar Ehdai	USA	Georg Salomon	Germany
Mark Emberton	UK	Peter Scardino	USA
Ferdinand Frauscher	Austria	Stephen Scionti	USA
Jurgen Futterer	The Netherlands	Ludger Sentker	Germany
Roman Ganzer	Germany	Martin Schostak	Germany
Sangeet Ghai	Canada	Chandru Sundaram	USA
Paolo Gontero	Italy	Joachim Thürhoff	Germany
Mitchell Gross	USA	John Trachtenberg	Canada
Rajan Gupta	USA	Matvey Tsivian	USA
Boris Hadaschik	Germany	Baris Turkbey	USA
Thomas Olivier Henkel	Germany	Osamu Ukimura	USA
Markus Hohenfellner	Germany	Massimo Valerio	Switzerland
Stephen Jones	USA	Arnauld Villers	France
Steven Joniau	Belgium	Jochen Walz	France
Christof Kastner	UK	John Ward	USA
Aaron Katz	USA	Hessel Wijkstra	The Netherlands
Laurence Klotz	Canada		
