# Supplementary material for: Standardization of definitions in focal therapy of prostate cancer: report from a Delphi consensus project
Source: World J Urol. 2016 Feb 18;34(10):1373–82. doi: 10.1007/s00345-016-1782-x (PMC5026990; doi:10.1007/s00345-016-1782-x)
Supplement: Supplementary file 2 — Supplementary material 2 (PDF 326 kb) [file 345_2016_1782_MOESM2_ESM.pdf]

Addendum 2. Results of Online rounds and consensus statements.

FT=Focal Therapy

Question	Response
**General Definitions**	
**Within the definition of targeted FT lies treating: (round 2)**	
**tumor + safety margin**	
• Yes	97%
• No	2%
• I don't know	2%
**one quadrant**	
• Yes	57%
• No	40%
• I don't know	3%
**one lobe (hemiablation)**	
• Yes	55%
• No	43%
• I don't know	2%
**both lobes sub-totally**	
• Yes	31%
• No	62%
• I don't know	7%
**Consensus statement after meeting:** The definition of targeted FT should be: a lesion-based focal treatment of the identified tumors plus a safety margin. Ablating a quadrant, a lobe or both lobes sub-totally is defined as FT	
**The aim of targeted FT in PCa should be defined as: (round 2)**	
• The eradication of all tumor	5%
• The eradication of all significant tumor	86%
• The eradication of the index lesion	7%
• Other	0%
• I don't know	2%
**Consensus statement after meeting:** The aim of both targeted FT and FT should be the eradication of all significant cancers.	
**What is the definition of subtotal ablation? (round 2)**	
• Any ablation where less than the whole gland is treated	84%
• An ablation where only one neurovascular bundle and a small portion of surrounding tissue is spared	3%
• An ablation where at least ¾ of the prostate is treated	7%
• Other	2%
• I don't know	3%
**Consensus statement after meeting:** The definition of subtotal ablation should be any ablation where less than the whole gland is treated.	
**What is the definition of extended-hemiablation? (round 2)**	
• An ablation where one lobe is completely treated plus a margin of the other lobe, regardless of shape	86%
• An ablation where one lobe is completely treated plus one quadrant of the other lobe (hockey stick)	5%
• Other	2%
• I don't know	7%
**Consensus statement after meeting:** An extended hemi-ablation is an ablation where one lobe is completely treated plus a margin of the other lobe regardless of shape	
**In targeted FT, which of the following best defines "index lesion"? (round 2)**	
• Highest Gleason score lesion determined by (targeted) biopsies	86%
• Largest lesion determined by imaging (and confirmed by biopsies)	7%
• Other	5%
• I don't know	2%

**Which of the following statements best characterizes the term index lesion? (round 3)**<br>• The index lesion is the single focus with the highest tumor grade and volume, where grade is more important<br>• The index lesions are all significant lesions that could lead to disease progression; it can be multiple lesions per patient<br>• Other<br>• I don't know<br><br>**Consensus statement after meeting:** The index lesion is the single dominant lesion in terms of grade and size where grade is more important. There can be only 1 index lesion, however the term index lesion itself may be of limited use in the context of FT. It is more important to have an overview of all significant lesions that need to be treated rather than a single defined index lesion.	60%<br>37%<br>1%<br>1%
**What is the definition of salvage FT: (round 2)**<br>• FT after any radical treatment<br>• FT after EBRT only<br>• FT after any treatment including previous FT<br>• Other<br><br>**What is the definition of salvage FT? (round 3)**<br>• FT after any radical treatment<br>• FT after any treatment including previous FT<br>• FT after any treatment including FT, except radical prostatectomy<br><br>**Consensus statement after meeting:** Salvage FT refers to the situation where FT is applied to the prostate after whole gland therapy, or in the same region of the prostate as previous FT. The prostate gland has to be in place.	43%<br>9%<br>43%<br>5%<br><br>36%<br>38%<br>26%
**Success and Failure in Focal Therapy**	
**When reporting ablation failure: (round 2)**<br>• Targeted biopsies of the target zone must confirm suspicion on imaging<br>• Positive Imaging inside target zone is enough to report "ablation failure"<br>• Other<br>• I don't know<br><br>**The best definition of ablation failure in focal therapy is: (round 3)**<br>• Tumor in the target zone<br>• Tumor in the target zone or directly adjacent to the target zone<br>• Clinically significant tumor in target zone<br>• Clinically significant tumor in target zone or directly adjacent to the target zone<br>• I don't know<br><br>**Consensus statement after meeting:** Ablation failure is a failure of the technique to destroy the tissue in the treated zone, evidenced by tumor found within the treated zone. Ablation failure is just one of the causes that can lead to failure of FT as a whole. Other types of failure include targeting failure and selection failure. Ablation failure must be confirmed by targeted biopsy.	86%<br>9%<br>5%<br>0%<br><br>55%<br>23%<br>9%<br>12%<br>1%
**Radiographic suspicion of ablation failure should be defined as: (round 2)**<br>• Imaging positive in treatment area<br>• Imaging positive anywhere in the prostate<br>• Other<br>• I don't know<br><br>**In defining "radiographic suspicion of ablation failure" following FT, the following imaging modalities can be used: (round 2)**<br>• mpMRI<br>• CEUS<br>• PET-scan<br>• Other<br>• I don't know<br><br>**Consensus statement after meeting:** The definition of radiographic suspicion of ablation failure is imaging suspect for tumor presence within the treated zone. MpMRI a suitable imaging modality to determine ablation failure.	89%<br>9%<br>2%<br>0%<br><br>100%<br>20%<br>16%<br>0%<br>0
**What is the definition of "residual disease"? (round 2)**<br>• Any tumor left anywhere in the prostate after FT<br>• Any tumor left in the target zone<br>• Other<br>• I don't know<br><br>**Consensus statement after meeting:** The definition of residual disease is: Cancer remaining in the target zone after FT.	11%<br>82%<br>5%<br>2%

**Selection failure in focal therapy in PCa is defined by: (Round 2)**	
**Significant disease in short-term follow-up biopsies outside ablation zone**	
• Yes	67%
• No	26%
• I don’t know	7%
**Significant disease in short-term follow-up biopsies inside ablation zone**	
• Yes	35%
• No	55%
• I don’t know	10%
**Identification of metastatic disease in short-term follow-up**	
• Yes	84%
• No	4%
• I don’t know	12%
**Identification of locally advanced disease in short-term follow-up**	
• Yes	88%
• No	3%
• I don’t know	9%
**The need for whole-gland treatment**	
• Yes	59%
• No	26%
• I don’t know	14%
**Consensus statement after meeting:** The definition of selection failure is: FT was inappropriately indicated, evidenced by short-term post-treatment identification of metastatic or locally advanced disease. There is no agreement on whether significant PCa in short-term biopsies taken inside or outside the treatment zone and the need for whole gland treatment during follow-up constitute selection failure.	
**The definition of biochemical progression after targeted FT should contain: (round 2)**	
• PSA	82%
• PCA3	0%
• phiPSA	2%
• Not possible in FT	14%
• Other	2%
• I dont’t know	0%
**The best PSA-based definition for biochemical progression following targeted FT is: (round 2)**	
• Phoenix criteria (nadir +2)	27%
• Original ASTRO criteria (3 consecutive rises above nadir)	2%
• Stuttgart criteria (nadir +1.2)	2%
• Nadir +2 AND PSA velocity of >0.75/y	4%
• PSA doubling time	4%
• Persistent PSA rise	7%
• PSA should not be used	7%
• Other	5%
• I don't know	43%
**Consensus statement after meeting:** PSA is the best marker to monitor disease after targeted FT. However there is currently no data on how to use PSA, i.e. there is no data to support any of the definitions for biochemical recurrence in the context of targeted FT.	
**What is the best definition for pathological progression? (round 2)**	
• Higher Gleason score than initial biopsy in any biopsy (inside or outside targeted zone)	9%
• Higher volume of disease than initial biopsy (number of positive cores or tumor involvement per core)	2%
• Both should be considered pathological progression	88%
• Other	0%
• I don’t know	2%
**Consensus statement after meeting:** The definition of pathological progression is an increase in Gleason score or tumor volume evidenced by a larger number of positive biopsies or larger per-core tumor involvement.	

Baseline and outcome functional measures	
**The definition of functional success of FT should contain: (round 1)**	
• Maintenance of voiding pattern	91%
• Maintenance of erectile function	10%
• Maintenance of Quality-of-life	0.0%
• No side effects	14%
• I don't know	3%
• Other	2%
**When should functional success of focal therapy be assessed? (round 2)**	
• After 3 months	5%
• After 6 months	18%
• After 1 year	70%
• After 2 years	4%
• After 3 years	0%
• After more than 3 years	2%
• Other	2%
**Consensus statement after meeting:** The definition of functional success of FT is the maintenance of voiding pattern, erectile function and Quality-of-Life assessed after 12 months.	
**While reporting potency in the context of FT, the definition should be based on: (round 3)**	
• A minimum IIEF-score	64%
• Ability to have sexual intercourse with or without PDE5i (PhosphoDiesterase-5 Inhibitors)	30%
• I don't know	6%
**The definition of significant erectile dysfunction is? (round 3)**	
• A IIEF-score below a certain threshold	65%
• Inability to have sexual intercourse	29%
• I don't know	6%
**In reporting significant deterioration of sexual function following FT, which definition should be used? (round 3)**	
• A minimum decrease in IIEF score of >5	17%
• Pre-Ft potency and post-FT impotency	74%
• I don't know	9%
**Consensus statement after meeting:** A qualitative definition of impotency exists: the persistent inability to attain and maintain an erection sufficient for satisfactory sexual performance. For reporting research the panel recommends defining significant erectile dysfunction using the IIEF-5 score < 21, determined at 1 year.	
**What is the definition of "sexually active": (round 1)**	
• Patient reported regular sexual intercourse	85%
• Other	3%
• I don't know	12%
**Consensus statement after meeting:** The definition of sexually active is based on patient-reported regular sexual activity.	
**What is the definition of urinary incontinence? (round 2)**	
• The use of pads (any number)	80%
• The use of pads or patient reported leakage	18%
• I don't know	2%
**What is the definition of urinary incontinence? (round 3)**	
• The use of pads (any number)	74%
• The use of pads or patient reported leakage	185
• Either the use of pads or reported leakage	9%
**In reporting significant deterioration of urinary function, what definition should be used? (round 3)**	
• An increase in IPSS score >5	78%
• Patient reported increased difficulty with voiding	6%
• It is best defined by a minimum increase in IPSS QoL score	4%
• Other	1%
• I don't know	10%

**Consensus statement after meeting:** The need to use pads or patient-reported leakage. More comprehensive data could be gathered by requesting patients to complete a micturition diary including the parameters: number of pads, leakage and urge.	
**The definition of (maintenance of) Quality-of-life should be based on: (Round 1)**<br><br>**UCLA-EPIC**<br>• Yes<br>• No<br>• I don't know<br><br>**EORTC QLQ-c-30**<br>• Yes<br>• No<br>• I don't know<br><br>**FACT-P and FACT-G**<br>• Yes<br>• No<br>• I don't know<br><br>**MAX-PC**<br>• Yes<br>• No<br>• I don't know<br><br>**Consensus statement after meeting:** A Quality-of-Life questionnaire should be used and both the UCLA-EPIC and the EORTC QLQ-c-30 tools can be used although neither one is validated for the specific context of focal therapy.	70%<br>10%<br>20%<br><br>75%<br>22%<br>19%<br><br>49%<br>22%<br>29%<br><br>12%<br>42%<br>46%
**Which of the following symptoms following FT constitute bowel toxicity/gastrointestinal (GI) side effects (round3)**<br><br>**Change in frequency**<br>• Yes<br>• No<br>• I don't know<br><br>**Soiling**<br>• Yes<br>• No<br>• I don't know<br><br>**Blood in stool**<br>• Yes<br>• No<br>• I don't know<br><br>**Mucus In stool**<br>• Yes<br>• No<br>• I don't know<br><br>**Fistula formation**<br>• Yes<br>• No<br>• I don't know<br><br>**Either of the above**<br>• Yes<br>• No<br>• I don't know<br><br>**Consensus statement after meeting:** The occurrence of: a change in stool frequency, fistula formation, soiling and/or blood in the stool after FT should constitute bowel toxicity/GI side effects. There is no consensus on whether mucus in the stool should also be included. The use of one of the existing grading systems for bowel toxicity is recommended.	87%<br>3%<br>10%<br><br>93%<br>3%<br>4%<br><br>88%<br>75<br>4%<br><br>78%<br>13%<br>9%<br><br>97%<br>1%<br>4%<br><br>75%<br>11%<br>14%

**The definition of intraoperative complications: (round 2)**<br><br>**Includes technical difficulties with equipment**<br>• Yes<br>• No<br>• I don't know<br><br>**Includes targeting difficulties due to anatomy**<br>• Yes<br>• No<br>• I don't know<br><br>**Includes complications that cause damage to the patients' health or require intervention to prevent this.**<br>• Yes<br>• No<br>• I don't know<br><br>**Consensus statement after meeting:** The definition of intraoperative complications includes only complications that cause damage to the patients' health or require intervention to prevent damage.	 <br><br>43%<br>55%<br>2%<br><br><br>41%<br>57%<br>2%<br><br><br>96%<br>0%<br>4%
**What is the definition of short-term side effects? Side effects that occur within: (round 2)**<br>• 1 week<br>• 2 weeks<br>• 30 days<br>• 6 weeks<br>• 3 months<br>• 6 months<br>• 1 year<br>• 1.5 years<br>• 2 years<br><br>**Consensus statement after meeting:** Short-term side effects are those that become apparent within 90 days after the procedure.	 4%<br>5%<br>25%<br>52%<br>11%<br>4%<br>0%<br>0%<br>2%
**What is the definition of serious side effects? (Round 2)**<br>• Clavien 2 or higher<br>• Clavien 3 or higher<br>• Other<br><br>**Consensus statement after meeting:** In defining the severity of side effects it is recommended to use the Clavien-Dindo-scale. The cut-off for a side effect being considered "serious" is 3.	 14%<br>89%<br>9%
**Procedural outcomes**	
**What is the definition of procedure time? (round 3)**<br><br>• The time the patient enters the OR until he leaves the operating room<br>• The time the treating physician can start (after anesthesia) until the treating physician is finished<br>• Other<br>• I don't know<br><br>**Consensus statement after meeting:** The definition of procedure time should be the time period starting after the anaesthetic induction is completed and the treating physician can start until the treating physician is finished.	 <br>13%<br>87%<br>0%<br>0%
**The definition of hospital stay is: (round 3)**<br>• From the day after the procedure until discharge<br>• From admittance until discharge<br>• From the day of the procedure until discharge<br><br>**Consensus statement after meeting:** The definition of hospital stay should be: the time from admittance until discharge.	 4%<br>11%<br>86%
**FT in day-care should be defined as: (round 2)**<br>• Admittance, treatment and discharge on the same day<br>• Admittance, treatment and discharge planned on the same day, although some patients spend the night<br>• Defined by OR schedule, irrespective of whether the patient stays the night<br><br>**Consensus statement after meeting:** The definition of focal therapy in day-care is: admittance, treatment and discharge on the same day.	 84%<br>13%<br>4%

**The definition of catheterization time is: (round 2)**<br>• The time form inserting the catheter until its removal, including time spent on the operating room and recovery unit<br>• The time form inserting the catheter until its removal, excluding time spent on the operating room and recovery unit<br>• The time form inserting the catheter post-operatively if necessary until its removal<br>• Other<br>• I don't know<br><br>**Consensus statement after meeting:** The definition of catheterization time should be the time from inserting the catheter until its removal, including time spent on the OR and the recovery-unit.	 81%<br>11%<br>7%<br>0%<br>0%
